# Effects of transportation on gastric pH and gastric ulceration in mares

**DOI:** 10.1111/jvim.15698

**Published:** 2020-02-03

**Authors:** Barbara Padalino, Georgina L. Davis, Sharanne L. Raidal

**Affiliations:** ^1^ Department of Agricultural and Food Sciences, Division of Animal Sciences Alma Mater Studiorum, University of Bologna Bologna Italy; ^2^ School of Animal and Veterinary Sciences Charles Sturt University Wagga Wagga New South Wales Australia

**Keywords:** alkaline reflux, bile salts, equine glandular gastric disease, equine squamous gastric disease, gastric emptying, gastric ulcer syndrome, gastrointestinal motility, volatile fatty acids

## Abstract

**Background:**

Transportation has been suggested as a risk factor for gastric ulceration in horses, but limited evidence supports this assumption.

**Animals:**

Twenty‐six Standardbred, Thoroughbred, and Warmblood mares from a university teaching herd.

**Methods:**

Twelve mares were confined for 12 hours, overnight, in reproductive stocks with indwelling nasogastric tubes (NGTs) to assess pH of gastric fluid (GF). Gastric ulceration was assessed endoscopically before and after confinement. Subsequently, 26 horses were transported for 12 hours, overnight, in 2 consignments. During transportation, GF was aspirated from indwelling NGT placed in the same 12 mares used in the confinement study, and gastric ulceration was assessed endoscopically before and after transportation in all horses.

**Results:**

The median pH of GF in confined horses was 1.70‐2.49 at each sampling point, and there was no apparent effect on gastric squamous ulcer scores. The median pH of GF from the same 12 horses at corresponding sampling times during transportation was 6.82‐7.22. Transportation was associated with increased gastric squamous ulcer scores, particularly in horses fasted for gastroscopy and NGT placement immediately before departure. Gastric emptying appeared delayed after transportation in horses fed before departure.

**Conclusions and Clinical Importance:**

Transportation is associated with increased gastric squamous ulceration and with increased pH of GF. These findings may be a consequence of impaired gastric emptying and reflux of alkaline small intestinal content, with factors such as duodenal bile salts and short‐chain fatty acids mediating mucosal injury.

**Abbreviations**
CK: creatine kinase
EGGD: equine glandular gastric disease
ESGD: equine squamous gastric disease
GF: gastric fluid
GI: gastrointestinal
LEL: lower explosive limit
NGT: nasogastric tube

## 1 INTRODUCTION

Gastric ulceration is the most common disease condition of the equine stomach^1^ and is associated with colic, decreased appetite, failure to thrive, and poor performance.^2^, ^3^ Equine gastric ulcer syndrome has been recommended as an encompassing term for all erosive and ulcerative conditions of the equine stomach.^4^ Importantly, however, given likely differences in the pathogenesis, clinical presentation and management of diseases of the squamous and glandular gastric mucosa,^5^, ^6^ the terms equine squamous gastric disease (ESGD) and equine glandular gastric disease (EGGD) are now preferred.^4^ The pathogenesis of ESGD is likely related predominantly to increased exposure to highly acidic gastric contents,^5^ although bile acids and volatile fatty acids also have been implicated in the induction of disease.^7^, ^8^, ^9^ Possible causes of EGGD are less well characterized although, in common with ESGD, it is hypothesized that erosion and ulceration occur as a result of an imbalance between aggressive and protective factors such that mucosal integrity is compromised. In horses, as in people with peptic ulcer disease, factors that decrease prostaglandin production^6^, ^10^ and possibly altered gastric microbiota^11^, ^12^ may contribute to the development of disease.

Horses travel frequently for performance, breeding, and other purposes. Transportation has been suggested as a risk factor for gastric ulceration^13^ and a recent survey of transport‐associated health problems indicated that gastrointestinal (GI) problems occur frequently in association with transportation of horses.^14^ Limited evidence currently is available regarding the effects of transportation on gastric ulceration or gastric pH.

Transportation is associated with factors that may contribute to gastric ulceration such as physiologic stress responses (increased heart rate and serum cortisol concentrations),^15^, ^16^, ^17^, ^18^, ^19^, ^20^, ^21^ changes in feeding practices and water consumption,^22^, ^23^ and changes in GI microbiota.^12^, ^24^ Our study was conducted to determine the impact of 12 hours of transportation without food or water on gastric ulcer scores and gastric fluid (GF) pH in horses. A secondary objective was to determine the effect of pretransport feeding practices on these outcomes. We hypothesized that transportation would be associated with ulceration of the squamous mucosa associated with acidic GF and that these effects would be more severe in horses fasted before transportation.

## 2 MATERIALS AND METHODS

### 2.1 Animals

Twenty‐six Standardbred (n = 14), Thoroughbred (n = 10), and Warmblood (n = 2) mares were included in the study. Mean age was 9.9 years (range, 4‐20 years), and mean body weight was 518.8 kg (range, 416‐658 kg). All horses were Charles Sturt University teaching or research horses and had been resident on site for ≥4 weeks. Prior transport history was unknown for each horse, although all had been transported on at least 1 prior occasion.

### 2.2 Experimental design

The study was conducted in 2 parts. Part 1 was conducted as a preliminary observational study to assess the effect of overnight confinement (1800‐0600 hours), without feeding, on gastric pH and gastric ulcer scores in 12 mares. Part 2 was conducted as an interventional study to determine the effect of overnight (1800‐0600 hours) transportation on gastric pH and gastric ulcer scores in 26 mares. Feed management before transportation consisted of feeding <60 minutes before departure (group 1, n = 7), feeding 6 hours before departure (group 2, n = 7) and fasting for 12 hours for gastroscopy and nasogastric tube (NGT) placement immediately before departure for the 12 mares used in study part 1. Study design is presented in Table 1, with further detail provided in Data S1 (Figure S1).

**Table 1:** Study design and treatment groups

			Treatment
Part 1 (confinement)	Day 1	Day 2	
	Horses	Horses	T0: Fasted 12 hours, gastroscopy, NGT; confine 12 hours
	H1‐H6 (n = 6)	H14‐H19 (n = 6)	T1: Repeat gastroscopy, remove NGT
	NGT	NGT	T3: (60 hours) Repeat gastroscopy
Part 2 (transport)	Trip 1	Trip 2	
	Day 15	Day 16	
	Horses	Horses	T0: Fasted 12 hours, gastroscopy, NGT; transport 12 hours
	H1‐H6 (n = 6)	H14‐H19 (n = 6)	T1: Repeat gastroscopy, remove NGT
	NGT	NGT	T3: (60 hours) Repeat gastroscopy
	H7‐H13 (n = 7) Group 1	H20‐H26 (n = 7) Group 2	T‐1: Gastroscopy 24 hours before transport Fed 1 hour pretransport (group 1, trip 1) Fed 6 hours pretransport (group 2, trip 2) Transport 12 hours T1: Repeat gastroscopy T3: (60 hours) Repeat gastroscopy
Age (years; mean, SD)	9.6 ± 4.2	7.4 ± 3.8	
Body weight (kg; mean, SD)	542.8 ± 77.7	548.2 ± 53.1	

Abbreviation: NGT, nasogastric tube.

#### 2.2.1 Part 1

Mares (n = 12) were confined in reproductive stocks (150 × 71 cm; height of front gate, 122 cm) as 2 groups, each of 6 horses, on consecutive nights. Horses were fed alfalfa hay (1%‐1.5% body weight) between 0600 and 0700 hours on the morning of confinement. Water was withheld from 1200 hours. Each horse underwent veterinary clinical examination and venous blood was collected for hematology, serum biochemistry, and blood gas analysis at 1400 hours, 4 hours before confinement (T0). Intestinal borborygmi were graded subjectively by one investigator (B.P.) based on auscultation of 4 abdominal quadrants (upper left, lower left, upper right, lower right) and recorded as 0 (no intestinal sounds auscultated in 60 seconds), 1 (decreased activity), 2 (normal activity, 2 or 3 discrete rumbling or gurgling noises in 30s), or 3 (increased activity) for each quadrant. These results were summed to give a GI activity score, as previously described.^19^ Horses were sedated (200 mg xylazine and 10 mg acetylpromazine, or 10 mg detomidine and 5 mg butorphanol IV) between 1600 and 1800 hours for gastroscopy and placement of an indwelling NGT (Veterinary Enteral Feeding Tube, 18 French × 250 cm; Mila International, Erlanger, Kentucky) and aspiration of GF. During confinement (1800‐0600 hours), horses were monitored continuously but were not offered food or water. GF was aspirated from the NGT every 2 hours. Clinical examination and venous blood collection were repeated at the end of confinement at 0600 hours the next day (T1), and after 8 hours (T2), and 60 hours (T3). Gastroscopy was performed at T1 and T3, with horses fasted for gastroscopy at T3 as before T0.

#### 2.2.2 Part 2

Effects of transportation were assessed in 26 horses transported as 2 consignments, each of 13 horses, on consecutive nights (trips 1 and 2, Table 1), completed 14 days after study part 1. Both trips were completed over an identical route covering approximately 880 km, with the same driver and vehicle, departing at 1800 hours and returning at 0600 hours the following morning. The transport vehicle was a 15‐horse trailer attached to a prime mover (LF290 18T, DAF Trucks Australia, Bayswater, Victoria, Australia). On each journey, 6 of the 12 horses used in part 1 of the study traveled in 6 bays, located at the back of the truck, which could be accessed continuously throughout the journey to permit aspiration of GF as during part 1 of the study. The remaining 7 horses were transported in compartments of the truck that could not be accessed during the journey. Horses traveling with NGT were fed between 0600 and 0700 hours on the morning of transportation, as before confinement in part 1. Water was removed at 1200 hours, with placement of the NGT during gastroscopic examination and aspiration of GF between 1600 and 1800 hours, as during part 1. Gastroscopy was performed on the 7 horses transported without NGT on the day before transportation, approximately 24 hours before departure (T‐1). Results from T‐1 were pooled with results from T0 for analysis of all pretransportation observations. Horses traveling without NGT were fed 2.5 kg of alfalfa hay within 1 hour of departure for trip 1 (horses 7‐13), or 6 hours before departure for trip 2 (horses 20‐27).

Clinical examination and venous blood collection were performed as in part 1 within 4 hours of departure and before sedation for gastroscopy and NGT placement (n = 12). Horses were monitored continuously during transit by video camera, with researchers (B.P., G.D.) traveling in the vehicle with access to the 6 horses with NGT during both trips. GF was collected every 2 hours from these horses. Clinical examination and venous blood collection were repeated for all horses at the end of transportation (T1), and after 8 hours (T2) and 60 hours (T3). Gastroscopy was repeated (n = 26) at T1 and T3, with horses fasted for gastroscopy at T3 as before T‐1 or T0.

### 2.3 Gastroscopy and GF pH determination

Endoscopic evaluation of the gastric mucosa was performed using a 3‐m endoscope (9 mm outer diameter, Olympus Medical Systems Corporation, Tokyo, Japan), as previously described,^25^ after insufflation of the stomach with air to permit complete visualization of the glandular and squamous gastric mucosa. Each horse's stomach was assessed using a validated equine scoring system^26^ with separate scores for the greater curvature, lesser curvature and fundus summed to give a squamous score, and separate scores similarly summed for fundic and pyloric glandular mucosa, as previously described.^25^ Gastroscopy examinations were video recorded and stored using a unique, randomly generated, 4‐digit identification number. Gastroscopy findings were scored in real time by 1 author (S.L.R.) and on review of deidentified video recordings by a second author (G.D.) blinded to time of sampling. Because findings were consistent for both methods of evaluation, real‐time results were analyzed because videos were missing or of inadequate quality for 11 examinations. Feed management before transportation influenced the amount of feed in the stomach at T1 (immediately after transportation). Consequently, on review of deidentified gastroscopy videos, gastric emptying was graded subjectively according to a scale developed as part of concurrent studies (Table 2).

**Table 2:** Feed retention score for subjective assessment of gastric emptying based on the amount of ingesta retained in the gastric lumen

Score	Description	Representative appearance	Endoscopic appearance
0	No retained gastric content, all glandular mucosa readily visualized		
1	Liquid content—glandular mucosa partly obscured by liquid content only	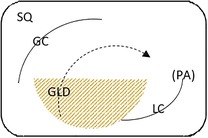	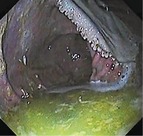
2	Slurry, no formed elements; glandular mucosa partly obscured by liquid with obvious particulate matter in suspension	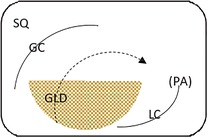	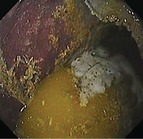
3	Feed material coalesced within the liquid slurry to form an “island” that is visible above the liquid horizon; glandular mucosa can be visualized with distension	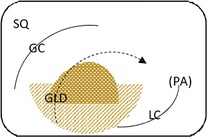	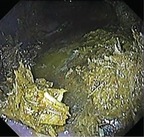
4	Feed material as a formed ball within the gastric lumen with minimal free fluid; glandular mucosa can be adequately visualized with distension and persistence	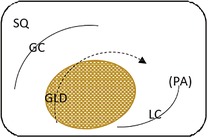	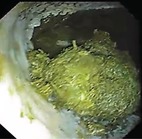
5	Large, solid feed ball in gastric lumen obstructing adequate visualization of glandular mucosa ± lesser curvature despite distension	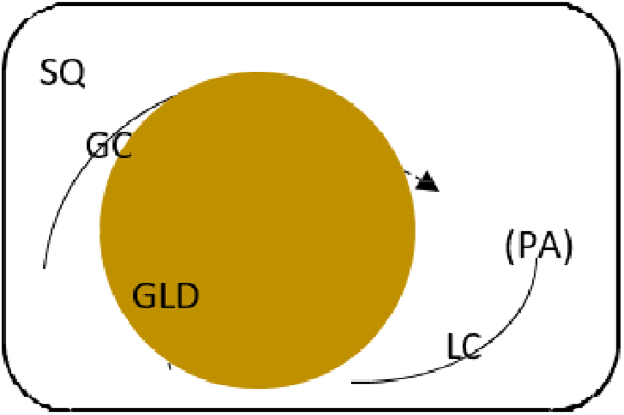	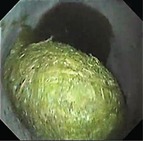

Gastric fluid was aspirated via the endoscope at the completion of mucosal scoring. Indwelling NGT were then placed by grasping a small piece of tape at the distal end of the NGT using endoscopic biopsy forceps and passing the 3‐m endoscope and NGT into the stomach, as previously described.^25^ GF (1‐10 mL) was aspirated every 2 hours via the NGT. Fluid was placed immediately into 50‐mL polypropylene containers, and pH was determined within 15 minutes of collection using a hand‐held pH meter (220 Portable, Temperature Calibrated pH/mV Meter; Instrument Choice, trading as Synotronics Pty Ltd, Regency Park, South Australia, Australia; pH range, 1‐14; 0.1 pH resolution; 0.2 pH accuracy). The probe was calibrated and verified against standard solutions of pH 7, 4, and 1 before sample analysis to ensure pH readings were within 0.1 of the expected pH for each standard solution.

### 2.4 Environmental parameters and air quality

During transportation, temperature (°C), humidity (%), heat index (°C), and wind speed (m/s) were monitored continuously using 3 weather trackers (Kestrel: 4000 Pocket Weather Tracker, Nielsen‐Kellerman, Boothwyn, Pennsylvania). The concentration of oxygen (O_2_, vol %), ammonia (NH_3_, ppm), hydrogen sulfide (H_2_S, ppm), carbon monoxide (CO, ppm), and methane (CH_4_, % of lower explosive limit [LEL]) were monitored by 3 gas detectors (Dräger X‐am 5000, Serial Number: ARFB0560, Dräger Safety AG & Co., Lübeck, Germany). Gas and weather trackers were fixed to the roof of the truck in each section of the trailer.

### 2.5 Hematology and serum biochemistry

Routine hematology parameters were determined using an automated cell counter analyzer (Cell Dyn 3700; Abbott, Chicago, IL). Total protein and albumin concentrations and creatine kinase (CK) and aspartate aminotransferase activities were measured in heparinized plasma samples using the Konelab 20XT photometer (Thermo Fisher Scientific, Finland, Europe). Plasma fibrinogen concentration was calculated by heat precipitation.^27^ Ionized sodium (Na^+^), potassium (K^+^), calcium (Ca^2+^), glucose, and lactate concentrations were measured in heparinized whole blood using a blood gas analyzer (Gem Premier 3500; Diamond Diagnostic, Holliston, Massachusetts). Plasma cortisol concentrations were measured by radioimmunoassay using the ImmunChem Cortisol 125 kit (MP Biomedicals, LLC, Orangenburb, New York).

### 2.6 Statistical analyses

Power analysis before the study and based on previous work conducted by our group suggested that 6 horses (the minimum subset of horses in the experimental design described above) afforded satisfactory power (>0.8, *α* = .05) to discriminate minimum differences in mean results for CK of 16.5 IU/L, cortisol of 0.5 mmol/L, and gastric pH of 2.2, assuming SD of 9.9 IU/L, 0.3 mmol/L, and 1.3 respectively. All data were evaluated initially using summary statistics. Differences between hematological and serum biochemistry parameters were compared by 2‐way repeated measures analysis of variance (ANOVA) after testing for normality using the D'Agostino and Pearson test. Where possible, nonparametric results were log‐transformed before analysis, or nonparametric tests were used. The effect of time of collection on pH of aspirated GF during confinement and transportation was determined by separate 1‐way ANOVA on ranks (Friedman test), with post hoc testing using Dunn's multiple pairwise comparisons. Comparisons between samples obtained from confined and transported horses were performed on log‐transformed data by 2‐way ANOVA with time, treatment and their interaction as fixed effects. During confinement and transportation, summed gastric ulcer scores were compared using Friedman and Dunn's tests. Comparisons between results from confined and transported horses at T0, T1, and T3 were made using separate Mann‐Whitney tests. The effects of time of feeding before departure on gastric feed retention score and on summed gastric ulcer scores were determined using the Kruskal‐Wallis test. For all statistical analyses, a *P* value of <.05 was used to indicate significance, and all analyses were performed using GraphPad Prism version 7.00 for Windows (GraphPad Software, La Jolla, California, http://www.graphpad.com).

## 3 RESULTS

### 3.1 Transportation

Noxious gases (H_2_S, CO, and CH_4_) remained below limits of detection (2 ppm, 2 ppm, and 1% LEL, respectively) at all times and in all locations during both trips. Oxygen was stable at 20.9% for the duration of both transport events. Ambient temperature ranged from 18.5°C to 5.8°C, relative humidity from 54% to 89% and wind speed from 0 to 2.5 m/s during trip 1, and from 14.5°C to 6.0°C, 59%‐85% and 0‐1.5 m/s, respectively, during trip 2; these results were consistent with meteorological observations for Wagga Wagga on the evenings when horses were confined (minimum temperatures of 8.1°C and 5.0°C).

**Figure 1:**
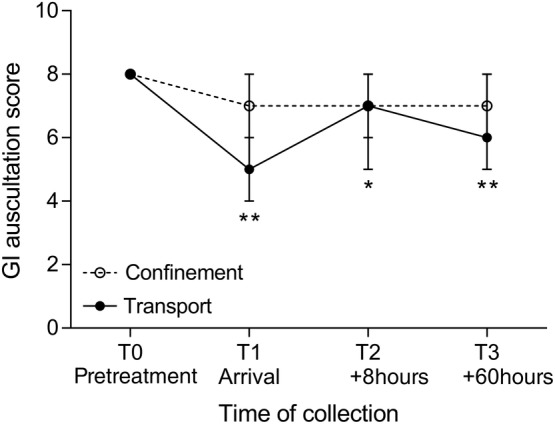
Effect confinement and transportation on gastrointestinal (GI) sounds assessed by abdominal auscultation. Results are presented as median (95% confidence interval) with differences within each treatment assessed by 1‐way analysis of variance on ranks using the Friedman test and significant differences at individual points compared with results at T0 determined by Dunn's multiple comparisons test (*P < .05; **P < 0.001). No significant time of collection effect was observed in confined horses

**Figure 2:**
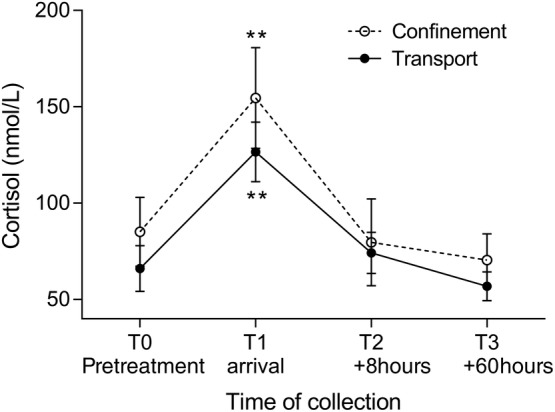
Effect confinement and transportation on plasma cortisol concentrations. Both interventions were significant (**P < .001), and the difference between transport and confinement was significant (*P* = .002), although interactions were not significant. Results are shown as mean and 95% confidence interval

All horses traveled well, with no clinical signs of injury or illness attributable to transportation. Transient, but significant (*P* < .001), increases in heart rate and rectal temperature were observed at T1, immediately on arrival for transported horses, but not observed at the corresponding time for horses during confinement only (Figure S1). Mean rectal temperature still was increased at T2, 8 hours after return, for transported horses. Body weight and respiratory rate showed no changes attributable to transportation or confinement. Abdominal auscultation identified decreased GI sounds at each examination after transportation (Figure 1), but no changes were observed in confined horses. Both transportation and confinement were associated with transient increases in plasma cortisol concentrations (Figure 2). Transportation was associated with increased white cell and neutrophil counts, and these changes were not observed in horses in association with confinement (Figure S2). A significant difference (*P* = .04) was evident for red cell count for transported horses in comparison with results during confinement, but pairwise comparisons were not significant. Transportation, but not confinement, was associated with increased plasma activity of CK, and plasma concentrations of protein, albumin, sodium, ionized calcium, lactate, and glucose (Figures S3‐S5). Plasma potassium concentrations varied with both transportation and confinement (Figure S4).

### 3.2 Effect of confinement on gastric ulceration and pH

**Figure 3:**
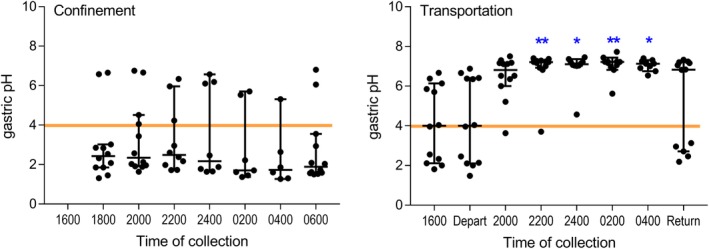
pH results for gastric fluid (GF) aspirated every 2 hours via indwelling nasogastric tubes from 12 horses during confinement (left) and transportation (right). Results are shown as median (horizontal bar) and range (whiskers), with all available data shown. Transportation, but not confinement, was associated with significantly increased pH of GF, with results significantly greater than obtained at departure shown (**P < .01; *P < .05)

Median pH of aspirated GF was 2.43 (range, 1.31‐6.65) before confinement. Results obtained from samples aspirated during confinement ranged from 1.27 to 6.80, with median values between 1.70 and 2.49 at each sampling point. No significant effect of time was observed on median pH of aspirated GF during confinement (*P* = .39, Figure 3). GF pH remained <4 overnight for most horses (Figure 3).

**Figure 4:**
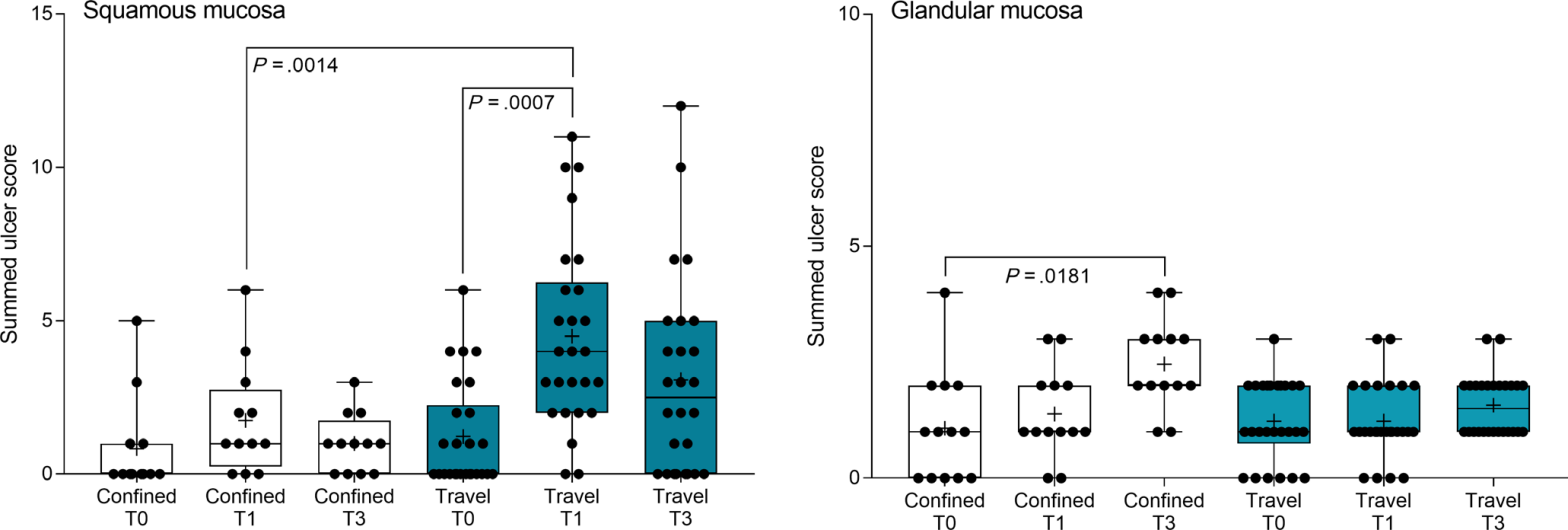
Summed squamous ulcer scores before (T0), immediately after (T1), and 60 hours after (T3) overnight confinement or transportation. Median squamous score increased after transportation (blue), but not after confinement (white), with significant differences observed between T0 and T1 for transported horses, and at T1 when transported horses were compared with confined horses. A minor effect on glandular ulcer score was observed in confined horses (white), but transportation had no effect on glandular ulcer scores (blue). Results are shown as median (horizontal bar), mean (cross), quartiles (box), and range (whiskers), with all data points shown. Significant differences are shown

Gastric emptying was satisfactory to permit full evaluation of squamous and glandular mucosa in all horses at T0 (before confinement), T1 (after 12 hours of confinement without feeding), and at T3 (60h after confinement). Summed gastric squamous ulcer scores ranged from 0 to 5 at T0, before confinement (median, 0), and increased by 1‐4 during confinement for 6 (of 12) horses (Figure 4). Two horses had grade 3 lesions of the lesser curvature after confinement, however summed squamous scores (median, 1; range, 0‐6) were not significantly affected by confinement (*P* = .08, Figure 4). A modest increase in median glandular score was observed at T3 (median, 2; range, 0‐4), compared to median glandular score at T0 (median, 1; range, 0‐4), but all lesions were mild (≤2) in the glandular mucosa of the fundus or pyloric antrum (Figure 4).

### 3.3 Effect of transportation on gastric ulceration and pH

Attempts were made to aspirate GF through the endoscope before departure (T0, n = 12) or on the day before transportation (T‐1, n = 14), and from all horses on return after transportation (T1, n = 26). Median pH of GF before transportation was 4.00 (range, 1.67‐6.67; n = 24); after transportation the median pH of endoscopically collected GF was 3.04 (range, 2.03‐7.33; n = 20). Endoscopic aspiration of GF was not possible from horses with substantial amounts of feed retained in their stomachs (feed retention of grades 3‐4), including 6 of 7 horses fed immediately (<1 hour) before departure. No significant difference was found in median pH of endoscopically aspirated GF from horses after transportation in comparison with results obtained before departure (*P* = .55).

Median pH of samples aspirated before departure from horses with indwelling NGT was 4.00 (range, 1.48‐6.88; n = 12). Median pH of GF samples aspirated during transportation was between 6.82 and 7.22, significantly higher than before transportation (*P* < .001, Figure 3). Comparison of log transformed results indicated that the pH of aspirated GF from transported horses at 2200 hours (*P* = .001), 2400 hours (*P* = .007), 0200 hours (*P* < .001), 0400 hours (*P* < .001), and 0600 hours (*P* = .015) was significantly increased when compared with corresponding results obtained during confinement. Median pH (6.83; range, 2.19‐7.33; n = 12) was increased on return for these horses, but a number of individual samples had much lower values at this time (Figure 3).

**Figure 5:**
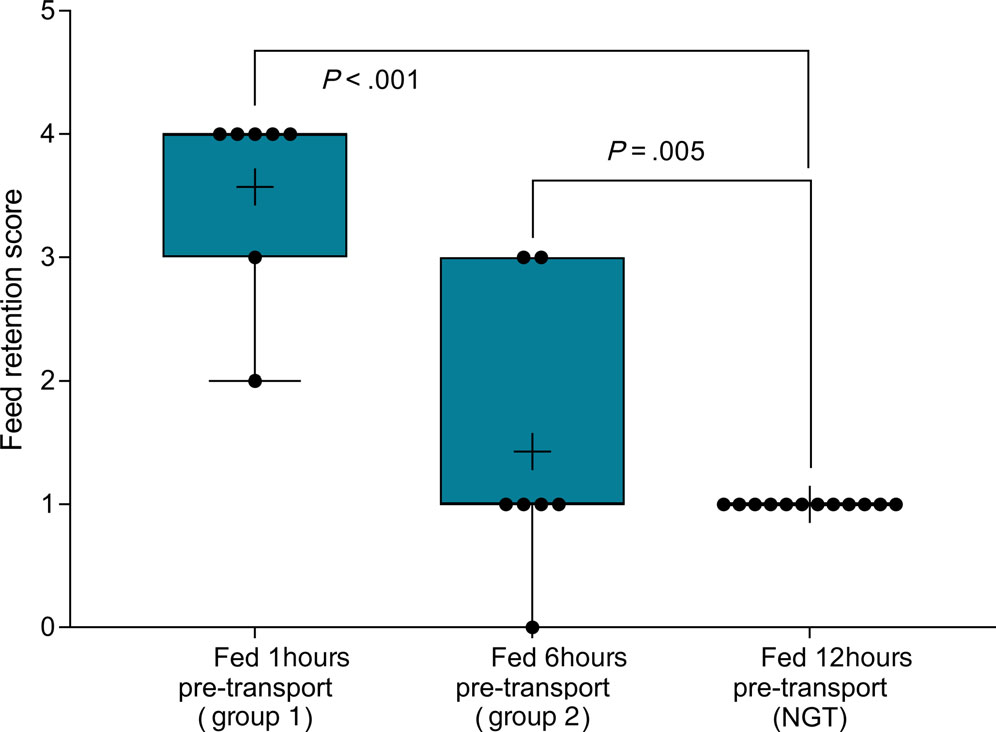
Effect of time of feeding before departure on feed retention scores at T1 (immediately after 12‐hour transportation). Median scores were increased in horses fasted for 1 or 6 hours before departure, in comparison with horses fasted for 12 hours. Results are shown as median (horizontal bar), mean (cross), quartiles (box) and range (whiskers), with all data points shown. Significant differences are shown

Gastric emptying was satisfactory to permit evaluation of squamous and glandular mucosa in all horses at T‐1 or T0 (before departure) and at T3 (60h after transportation). However, gastric feed retention at T1 (immediately after transportation) varied according to the time of feeding before departure (Figure 5), with horses fed immediately (1 hour) or 6 hours before transportation having significantly more ingesta retained in their stomachs. Glandular fundic mucosa was partially obscured for some group 1 horses (fed <1 hour before departure) at T1.

**Figure 6:**
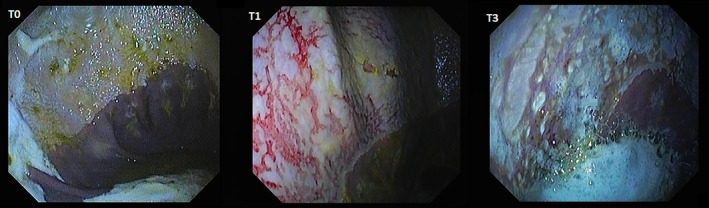
Representative images showing gastric ulceration in a transported horse at T0 (before departure), T1 (immediately on return from transportation) and T3 (60 hours after transportation). Ulceration was attributed to splashing of liquid content against the gastric mucosa, and was particularly marked in horses fasted before transportation for placement of indwelling nasogastric tubes. All images show the greater curvature of the stomach. At T0 there is hyperkeratosis only (grade 1). At T1 there is extensive ulceration extending from the margo plicatus, with bleeding evident in some affected areas (grade 4 of greater curvature and fundus). By T3 the affected area appears less inflamed and ulcers are no longer bleeding; however, extensive ulceration is still apparent, and remains deep in some affected areas (grade 4)

**Figure 7:**
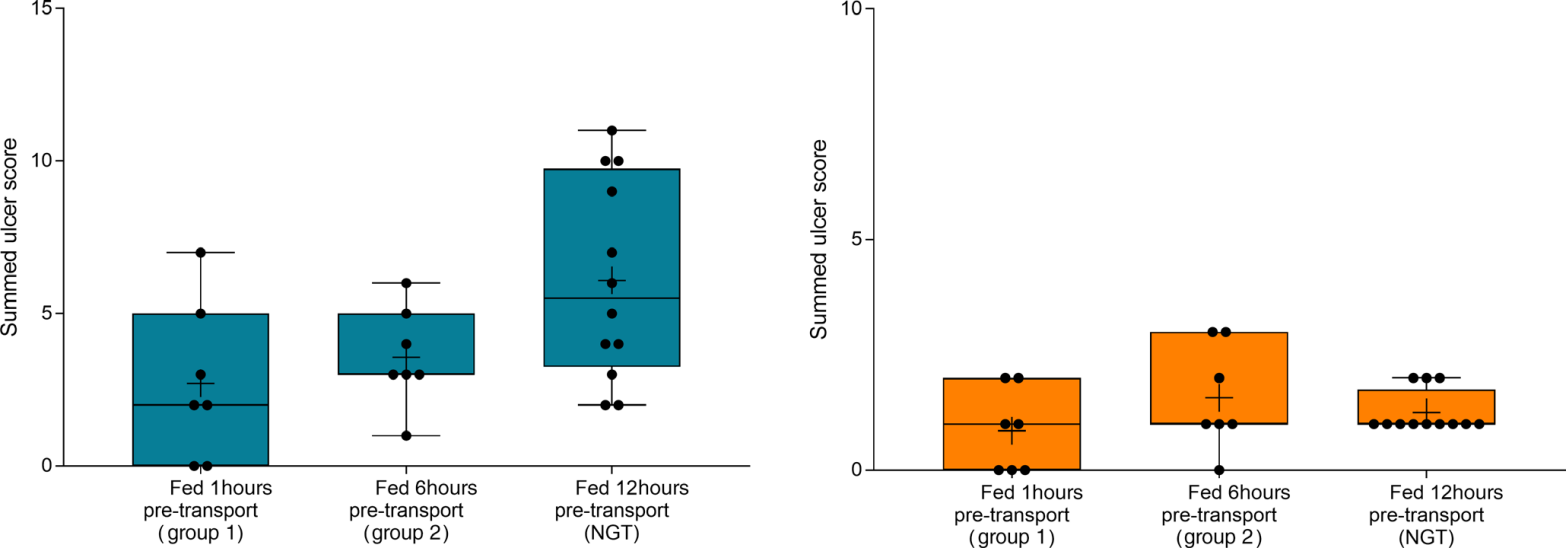
Gastric ulcer scores in horses according to time of feeding before transportation. Horses fasted for 12 hours before placement of nasogastric tubes had higher summed squamous scores (blue) than horses that were fed at 1200 hours (6 hours before departure) or at 1700 hours (immediately before departure), but observed differences were not significant (*P* = .06). No effect was observed on glandular ulcer scores (orange). Results are shown as median (horizontal bar), mean (cross), quartiles (box), and range (whiskers), with all data points shown

Summed gastric squamous ulcer scores ranged from 0 to 6 (median, 0) in horses before transportation (n = 26), and were higher after transportation for 15 (of 26 horses), including 10 (of 12) horses with indwelling NGT. Median squamous ulcer score was significantly (*P* < .001) higher after transportation (Figure 4). Severe ulceration (grade 4) was evident at the margo plicatus (greater and lesser curvatures) and extending to involve the fundic squamous mucosa in some horses after transportation (Figure 6), and appeared to be associated with contact between GF and the squamous mucosa, as evidenced by particulate content and bile staining of the mucosa in affected horses. Subjectively, the severity of squamous ulceration appeared to be inversely related to the amount of feed within the stomach during transportation, whereby more marked feed retention was associated with less ulceration (Figure 7). Transportation or feeding did not affect summed scores for the glandular mucosa.

Squamous ulcer scores improved spontaneously for 12 horses by T3, but 6 horses had increased ulcer scores at this time compared to T1. The majority of fundic squamous lesions had improved dramatically without treatment, but lesions of the lesser curvature had not resolved for many horses, and 9 (of 26) horses were treated with omeprazole (1 mg/kg PO q24h for 7 days; Gastrozol Daily Oral Paste for Horses; Virbac [Australia] Pty Ltd, Milperra, New South Wales, Australia) on completion of the study to ensure resolution of squamous lesions of ≥3 grade severity in ≥1 locations (Supplementary data, Figure S6).

## 4 DISCUSSION

We evaluated the effect of transportation on gastric ulceration and pH of GF in horses. In part 1, a preliminary study was conducted in which horses were subjected to confinement, sampling and gastroscopy procedures that mimicked those experienced during transportation. Transported horses had well‐characterized clinical, hematological, and blood biochemistry changes reported after transportation in previous studies,^16^, ^19^, ^20^, ^28^, ^29^, ^30^, ^31^ and these changes were not observed in horses confined for a similar duration. Plasma cortisol concentrations increased in transported horses, as has been reported previously.^19^, ^21^, ^32^, ^33^ However, study design may have prevented discrimination of effects of transportation on cortisol because confinement was associated with a similar increase in plasma cortisol concentration and these results might reflect expected diurnal variations.^34^, ^35^ Indeed, observed changes were remarkably consistent with diurnal changes in plasma cortisol concentration reported previously.^35^

Our findings suggest that transportation of 12 hours without feeding may contribute to the development of ESGD because travel induced or exacerbated ulceration of the squamous mucosa in the majority of horses and was associated with a significant increase in squamous ulcer scores, particularly in horses fasted for 12 hours before transportation. To our knowledge, development of gastric squamous ulcers in such a short time frame (12 hours) has not been reported previously in horses. Squamous epithelial lesions in transported horses were associated with increased pH of GF (median values of 6.8‐7.2) in horses where indwelling NGT permitted aspiration of GF throughout the journey, a finding contradictory to our research hypothesis, which postulated that prolonged exposure to gastric acid might contribute to ESGD. During confinement, horses had GF of low pH for the duration of study part 1 (median values of 1.7‐2.49), as expected, and these horses did not have increased gastric squamous ulcer scores. Partitioning of gastric content may influence pH determination in horses,^36^, ^37^ but gastric emptying was similarly complete for horses with indwelling NGT after both confinement and transportation (T1). In the nearly complete absence of retained ingesta, aspiration of GF through the NGT and endoscope was relatively simple, and no differences were observed between samples obtained simultaneously via either NGT or endoscope, including endoscopically observed aspiration through the NGT, suggesting no partitioning of gastric content occurred. Divergent findings in confined and transported horses suggest that the observed effects on gastric pH cannot be attributed to the NGT.

Because the fundic squamous mucosa was discolored and had residual feed particles adherent after transportation in many horses, it was assumed that contact with GF contributed to mucosal damage. Lesions extending into the squamous mucosa from the margo plicatus are uncommon in spontaneous disease.^38^ These observations contradict a recent review emphazing the role of HCl as the dominant erosive agent in the development of ESGD^5^ and suggest that less acidic factors, such as short‐chain fatty acids^8^, ^9^ and duodenal bile salts,^7^ are important contributors to mucosal damage in some circumstances. Reflux of alkaline small intestinal content is observed commonly during gastroscopic examination, and samples obtained from NGT in our study were typically yellow, consistent with bile content. These observations are important because gastric ulcer prophylaxis before transportation using proton pump inhibitors such as omeprazole^39^ will not be effective if gastric acid is not mediating mucosal injury.

Increased pH was not identified in endoscopically derived GF samples in our study from horses fed before departure. Because endoscopic examination of 13 horses took approximately 90‐120 minutes to complete, and horses with NGT were sampled first, this observation was attributed to rapid return of gastric function after transportation, as was apparent on assessment of individual results from horses transported with NGT (Figure 3). Alternatively, retention of feed within the gastric lumen could have stimulated continued secretion of gastric acid in horses fed within 1 or 6 hours of departure.

Feed management before transportation affected gastric content observed in horses after transportation and appeared to influence the severity of squamous ulceration observed at this time, with an incremental effect on squamous ulcer scores associated with increased time between feeding and departure. The presence of feed in the stomach may be protective against the development of ESGD,^40^ by absorbing gastric secretions or duodenal reflux, and minimizing contact between the squamous mucosa and liquid content. Horses fasted for 12 hours before departure for gastroscopy and NGT placement had complete gastric emptying after transport with moderate amounts of liquid gastric content, whereas horses fed 6 hours before departure (group 2) or within 1 hour (group 1) of departure had incrementally more ingesta within their stomachs after transportation and small volumes of GF in the most central part of the glandular fundus only. Assuming transported horses were fasted at least 12 hours (group 1, fed <1 hour before departure) and up to 18 hours (group 2, fed 6 hours before departure), this observation suggests decreased gastric emptying relative to our observations at T0 and T3, when all horses had complete gastric emptying after 12 hours fasting. Abdominal auscultation also suggested decreased GI motility in transported horses, as has been reported previously,^19^ but decreased GI motility was not observed after confinement. Decreased GI motility is consistent with well‐characterized sympathoadrenal neuroendocrine responses to transportation^16^, ^21^, ^33^ and GI problems, notably colic and colitis, are commonly associated with transportation of horses.^14^, ^41^, ^42^ Road transportation has been recently determined to influence fecal microbiome^43^ and metabolic responses to diet and exercise.^44^ Together these findings suggest that transportation may predispose to GI disease by affecting GI motility. Further study is required to better characterize this effect, and to determine optimal feeding strategies before and during transportation.

Although treatment with omeprazole is unlikely to have prevented development of gastric ulceration in our study, several horses required omeprazole treatment to ensure resolution of ESGD, with lesions of the lesser curvature seemingly more refractive to healing, as has been reported previously.^45^ Lesions of the greater curvature and fundus, areas that are unlikely to experience ongoing contact with acidic gastric content after transportation, appeared to heal rapidly when horses were kept on pasture between T1 and T3 in our study. No effect of transportation, feeding or confinement was observed on glandular ulcer scores in our study.

Limitations of our study relate to the need to manipulate feeding practices before departure in order to place NGT for sampling of gastric content during transportation. For this reason, and because of limited access to some compartments of the transport vehicle during travel, aspiration of GF during the journey was possible for only 12 horses. However, this number was approximately twice the required number based on power analysis, and a clear effect of transportation, relative to confinement, was observed in horses with NGT. The study design also allowed assessment of changes in horses after different feeding practices and without the presence of the NGT. Although the macroscopic appearance of aspirated GF was consistent with refluxed small intestinal content, and changes were not observed in horses confined with indwelling NGT, we cannot definitively exclude the possibility that the NGT influenced GF pH, for example by stimulating excessive salivation. Further studies therefore are required to determine the generalizability of these findings, and to assess the effects of other management strategies, such as provision of water during transportation. Our findings suggest that the pH of GF might rapidly decrease on cessation of transportation, and future studies should control for delayed aspiration of GF at the end of transportation. It was not possible to randomize the order of treatment (confinement or transportation) in horses fitted with indwelling NGT, nor to randomize feeding before transportation (all group 1 horses were allocated to trip 1, and all group 2 horses to trip 2). Subjective grading of gastric ulcer scores, gastric feed retention and abdominal auscultation are inherent limitations of our study. The validity of endoscopic findings was protected by evaluation of deidentified video recordings, but assessors were not blinded during assessment of GI motility when completing physical examination of horses. Feed retention limited visualization of fundic glandular mucosa for some horses, although squamous and pyloric mucosa was satisfactorily visualized. The study was not specifically designed to evaluate the effect of transportation on GI motility, and effects of confinement on gastric emptying were indirectly assessed, without variation of feeding practices before confinement. Further studies are warranted therefore to better characterize possible effects of transportation on GI motility.

To our knowledge, our study is the first to unequivocally identify gastric ulceration associated with transportation of fasted horses, and therefore to support transportation as a risk factor for the development of ESGD. To our knowledge, rapid development (within 12 hours) of squamous ulceration has not been reported previously. The severity of changes, particularly in horses fasted before travel, suggests that horses should have access to feed until the time of departure, and potentially during transport, to limit contact between the squamous mucosa and gastric secretions or small intestinal reflux. Unexpectedly, gastric ulceration was associated with increased pH of GF during transportation, consequently the administration of proton pump inhibitors or histamine receptor antagonists to horses before transportation cannot be supported based on our findings. Although spontaneous recovery occurred in a number of horses, 9 (of 26) horses were treated with omeprazole after completion of the study, primarily to ensure healing of ulcerative lesions of the squamous mucosa at the lesser curvature. Study findings suggested that GI motility was decreased during transportation, but further studies are warranted to better characterize possible effects of transportation on GI motility, to ascertain whether our findings in mares are generalizable also to male horses and to determine whether feeding before departure is protective against development of gastric ulceration.

## CONFLICT OF INTEREST DECLARATION

Authors declare no conflict of interest.

## OFF‐LABEL ANTIMICROBIAL DECLARATION

Authors declare no off‐label use of antimicrobials.

## INSTITUTIONAL ANIMAL CARE AND USE COMMITTEE (IACUC) OR OTHER APPROVAL DECLARATION

This project was approved by the Charles Sturt University Animal Care and Ethics Committee (approval number A17011).

## HUMAN ETHICS APPROVAL DECLARATION

Authors declare human ethics approval was not needed for this study.

## Supporting information


**Data S1:** Details of study design
**Figure S1.** Experimental design. Horses were recruited in 2 groups, each of 13 mares. From each group, a subset of 6 mares was randomly selected for a preliminary study to determine the effect of overnight (12 hours) confinement on gastric ulceration and pH of gastric fluid. Confinement occurred on 2 consecutive nights, with the 6 horses from each group confined separately according to available facilities. Prior to confinement, gastric squamous and mucosal ulceration was assessed by gastroscopy, and indwelling nasogastric tubes (NGT) were placed to allow sampling of fluid every 2 hours during the night. After a 14‐day break, the effects of transportation were assessed in 2 identical trips on consecutive nights. The 12 horses that underwent confinement were transported (6 horses in each consignment) with indwelling NGT in place. Sampling was identical for these 12 horses before confinement and transportation: all horses were fasted for 12 hours prior to gastroscopy and placement of NGT before confinement or transportation. In order to assess the effect of feeding prior to transportation, remaining horses in group 1 (n = 7, trip 1) were fed <60 minutes before transportation, and remaining group 2 horses (n = 7, trip 2) were fed 6 hours before departure. Of necessity (to allow feeding on the day of transportation), gastroscopy of these 14 horses was performed the day before departure (T‐1), and these horses did not travel with indwelling NGT. Pretransportation gastroscopy findings at T‐1 and T0 were combined for analysis. Clinical examination and venous blood collection were performed for all horses prior to confinement / transportation (T0), and repeated on completion of 12 hours (overnight) confinement / transportation at 0600h (T1). Gastroscopy was repeated for all horses on completion of confinement / transportation (T1), and took approximately 120 minutes to complete for all 13 horses following transportation. Gastric fluid was aspirated from indwelling NGT during confinement / transportation and on arrival for the 12 horses with NGT; gastric fluid was collected from remaining horses at the time of gastroscopy. Clinical examination and venous blood sampling from all horses were repeated 8 hours following the end of confinement / transportation (T2), and again 60 hours following the end of confinement / transportation (T3). Horses were fasted for 12 hours gastroscopy at T‐1, T0 and T3. At T1 horses had been fasted for 24 hours (horses with NGT), approximately 18 hours (group 2 horses fed 6h prior to departure) or 12 hours (group 1 horses fed <60 minutes prior to departure). G/scope, gastroscopy; GF, gastric fluid; q2h, every 2 hours
**Figure S2:** Effects of confinement and transportation on body weight, heart rate, respiratory rate and rectal temperature. Time: treatment interactions were significant for heart rate (*P* < .001) and rectal temperature (*P* = .005). Significant time of collection effects associated with transportation are shown (*P < .01; **P < .001); *P* values are provided for significant differences between transportation and confinement at individual time points. Results are presented as mean (95% CI). No significant time of collection effects were observed in confined horses
**Figure S3:** Effect confinement and transportation on hematology findings. Within each treatment, significant time of collection effects are shown (*P < .05; **P < .001). Peripheral blood neutrophil counts were increased relative to pretransportation (T0) values on arrival (T1, *P* < .001) and at 12‐hour postarrival (T2, *P* < .001), but had returned to pretransportation values by 36‐hours posttransportation. Results obtained at T1 (*P* < .001) and T2 (*P* = .02) following transportation were also significantly greater than corresponding values during confinement. There were significant changes in peripheral lymphocyte counts immediately following both confinement and transportation, but there were no significant time of collection effects on red cell count during either intervention. Significant differences between transportation and confinement were evident at T1 for white cell (*P* = .009) and neutrophil counts (*P* < .001), and at T2 for neutrophils (*P* = .015). No significant time of collection effects were observed on red cell numbers. Results are shown as mean and 95%CI
**Figure S4:** Effect confinement and transportation on plasma creatinine kinase, protein and albumin concentrations. Significant effects within each treatment are shown (*P < .05; **P < .005); *P* values refer to significant differences between transportation and confinement at specific time points. Results are shown as median and 95%CI for CK, and were log transformed for statistical analyses. Other results are presented as mean and 95% CI
**Figure S5:** Sodium, potassium and calcium ion concentrations associated with transportation and confinement. Significantly differences in postintervention results are shown (*P < .05; **P<.001). Sodium results are shown as median and 95% CI, and analyses were performed on log transformed data; other results are shown as mean and 95% CI. Plasma sodium increased slightly immediately after transportation and decreased subsequently such that differences observed at T2 and T3 were significantly less than observed as T1, as shown. Changes observed with confinement were not significant, and differences between confined and transported horses were not different at any time. Plasma potassium ion concentrations changed with time during both transportation and confinement, with significant differences between T1 and subsequent samples as shown. Differences in plasma potassium concentrations between transported and confined horses at T2 were significant (*P* = .003). Plasma ionized calcium concentrations increased following transportation, but did not change associated with confinement. Differences between transported and confined horses were not significant at any time
**Figure S6:** Plasma glucose and lactate concentrations associated with transportation and confinement. Significant differences between pre‐ and post‐transport values are shown (**P < .001), and significant differences between T1 and T2, and between T1 and T3 are indicated. Differences in plasma lactate concentration in transported horses were different to those obtained associated with confinement at all time points (all *P* < .001), and differences in plasma glucose concentration between transported and confined horses were significant at T1. Results are shown as median and 95% CI, and analyses were performed on log transformed data
**Figure S7:** Grade 4 ulceration of the lesser curvature in two horses at T3, 60 hours following transportation
