# Supplementary material for: Effects of transportation on gastric pH and gastric ulceration in mares
Source: J Vet Intern Med. 2020 Feb 3;34(2):922–32. doi: 10.1111/jvim.15698 (PMC7096603; doi:10.1111/jvim.15698)

Figure S1: Experimental design. Horses were recruited in two groups, each of 13 mares. From each group, a subset of six mares was randomly selected for a preliminary study to determine the effect of overnight (12h) confinement on gastric ulceration and pH of gastric fluid. Confinement occurred on two consecutive nights, with the six horses from each group confined separately according to available facilities. Prior to confinement, gastric squamous and mucosal ulceration was assessed by gastroscopy, and indwelling nasogastric tubes (NGT) were placed to allow sampling of fluid every 2h during the night. After a 14 day break, the effects of transportation were assessed in two identical trips on consecutive nights. The twelve horses that underwent confinement were transported (six horses in each consignment) with indwelling NGT in place. Sampling was identical for these twelve horses prior to confinement and transportation: all horses were fasted for 12h prior to gastroscopy and placement of NGT prior to confinement or transportation. In order to assess the effect of feeding prior to transportation, remaining horses in Group 1 (n=7, trip 1) were fed <60 minutes prior to transportation, and remaining Group 2 horses (n=7, trip 2) were fed 6h prior to departure. Of necessity (to allow feeding on the day of transportation), gastroscopy of these 14 horses was performed the day prior to departure, and these horses did not travel with indwelling NGT. Pre-transportation gastroscopy findings at T-1 and T0 were combined for analysis. Clinical examination and venous blood collection were performed for all horses prior to confinement / transportation (T0), and repeated on completion of 12h (overnight) confinement / transportation at 0600h (T1). Gastroscopy was repeated for all horses on completion of confinement / transportation (T1), and took approximately 120 minutes to complete for all 13 horses following transportation. Gastric fluid was aspirated from indwelling NGT during confinement / transportation and on arrival for the 12 horses with NGT; gastric fluid was collected from remaining horses at the time of gastroscopy. Clinical examination and venous blood sampling from all horses were repeated 8h following the end of confinement / transportation (T2), and again 60h following the end of confinement / transportation (T3). Horses were fasted for 12h prior to gastroscopy at T-1, T0 and T3. At T1 horses had been fasted for 24h (horses with NGT), approximately 18h (Group 2 horses fed 6h prior to departure) or 12h (Group 1 horses fed <60 min prior to departure). G/scope, gastroscopy; GF, gastric fluid; q2h, every 2h.

	Day:	(-1)	0					1			2	3
	Samples:	T-1 (pre)				T0 (pre)			T1 (end)	T2 (+8h)	Rest day	T3 (+60h)
Horses	Time:	1600h	0600h	1200h	1600h	1700h	1800h		0600h	1400h		1800h
Group 1	H1, H2, H3, H4, H5, H6		Feed	Fast ~12h	G/scope, place NGT		Confined 12h - asp GF q2h (overnight)		G/scope, remove NGT			G/scope
	H7, H8, H9, H10, H11, H12, H13											
Group 2	H14, H15, H16, H17, H18, H19		Feed	Fast ~12h	G/scope, place NGT		Confined 12h - asp GF q2h (overnight)		G/scope, remove NGT			G/scope
	H20, H21, H22, H23, H24, H25, H26											
Rest 14 days												
Group 1 (trip 1)	H1, H2, H3, H4, H5, H6		Feed	Fast ~12h	G/scope, place NGT		TRANSPORT 12h - asp GF q2h (overnight)		G/scope, remove NGT			G/scope
	H7, H8, H9, H10, H11, H12, H13	G/scope (24h pre)				Feed	TRANSPORT 12h (overnight)		G/scope			G/scope
Group 2 (trip 2)	H14, H15, H16, H17, H18, H19		Feed	Fast ~12h	G/scope, place NGT		TRANSPORT 12h - asp GF q2h (overnight)		G/scope, remove NGT			G/scope
	H20, H21, H22, H23, H24, H25, H26	G/scope (24h pre)		Feed	Fast ~6h		TRANSPORT 12h (overnight)		G/scope			G/scope

Figure S2: Effects of confinement and transportation on body weight, heart rate, respiratory rate and rectal temperature. Time:treatment interactions were significant for heart rate (P<0.001) and rectal temperature (P=0.005). Significant time of collection effects associated with transportation are shown (*, P<0.01; **, P<0.001); P values are provided for significant differences between transportation and confinement at individual time points. Results are presented as mean (95% CI). No significant time of collection effects were observed in confined horses.

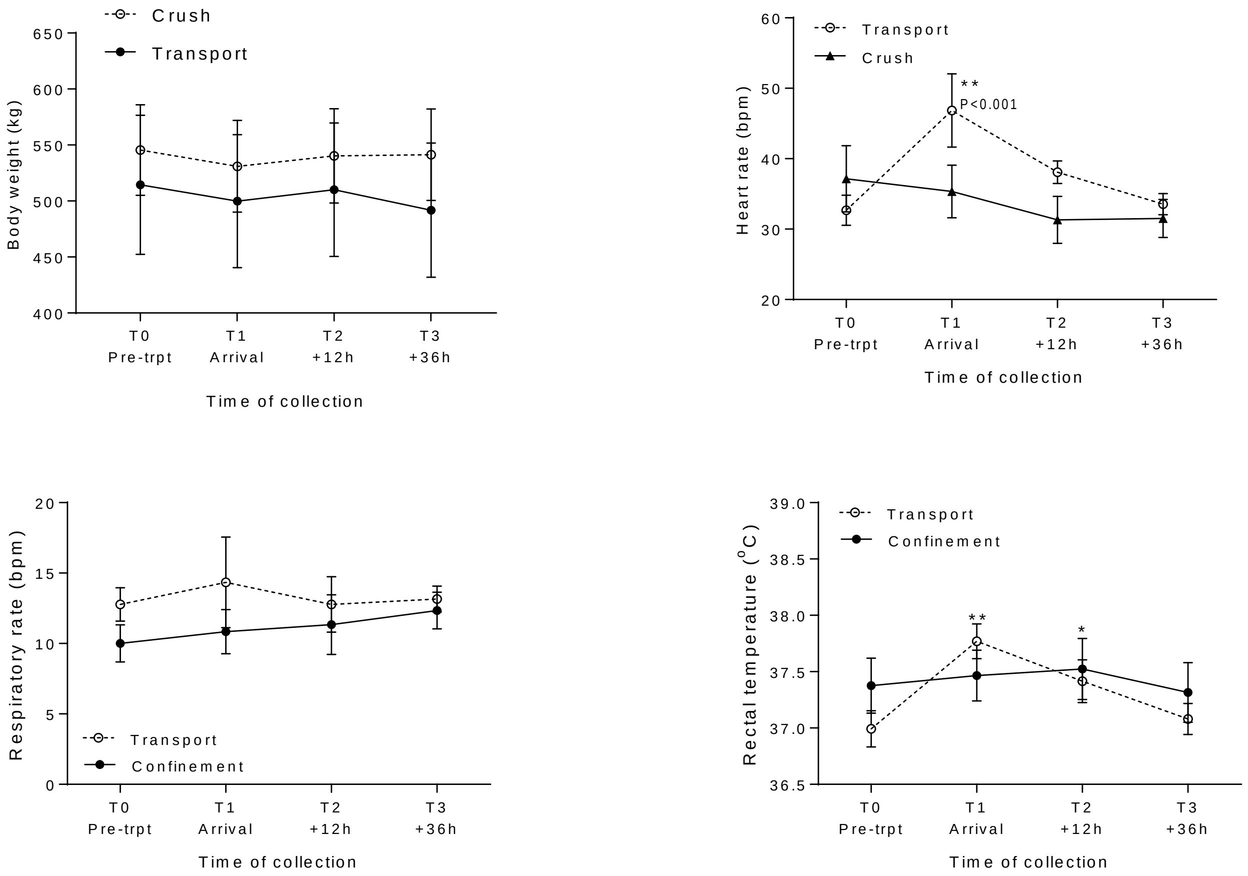

Figure S3: Effect confinement and transportation on haematology findings. Within each treatment, significant time of collection effects are shown (*, P<0.05; **, P<0.001). Peripheral blood neutrophil counts were increased relative to pre-transportation (T0) values on arrival (T1, P<0.001) and at 12h post-arrival (T2, P<0.001), but had returned to pre-transportation values by 36h post-transportation. Results obtained at T1 (P<0.001) and T2 (P=0.015) following transportation were also significantly greater than corresponding values during confinement. There were significant changes in peripheral lymphocyte counts immediately following both confinement and transportation, but there were no significant time of collection effects on red cell count during either intervention. Significant differences between transportation and confinement were evident at T1 for white cell (P=0.009) and neutrophil counts (P<0.001), and at T2 for neutrophils (P=0.015). No significant time of collection effects were observed on red cell numbers. Results are shown as mean and 95%CI.

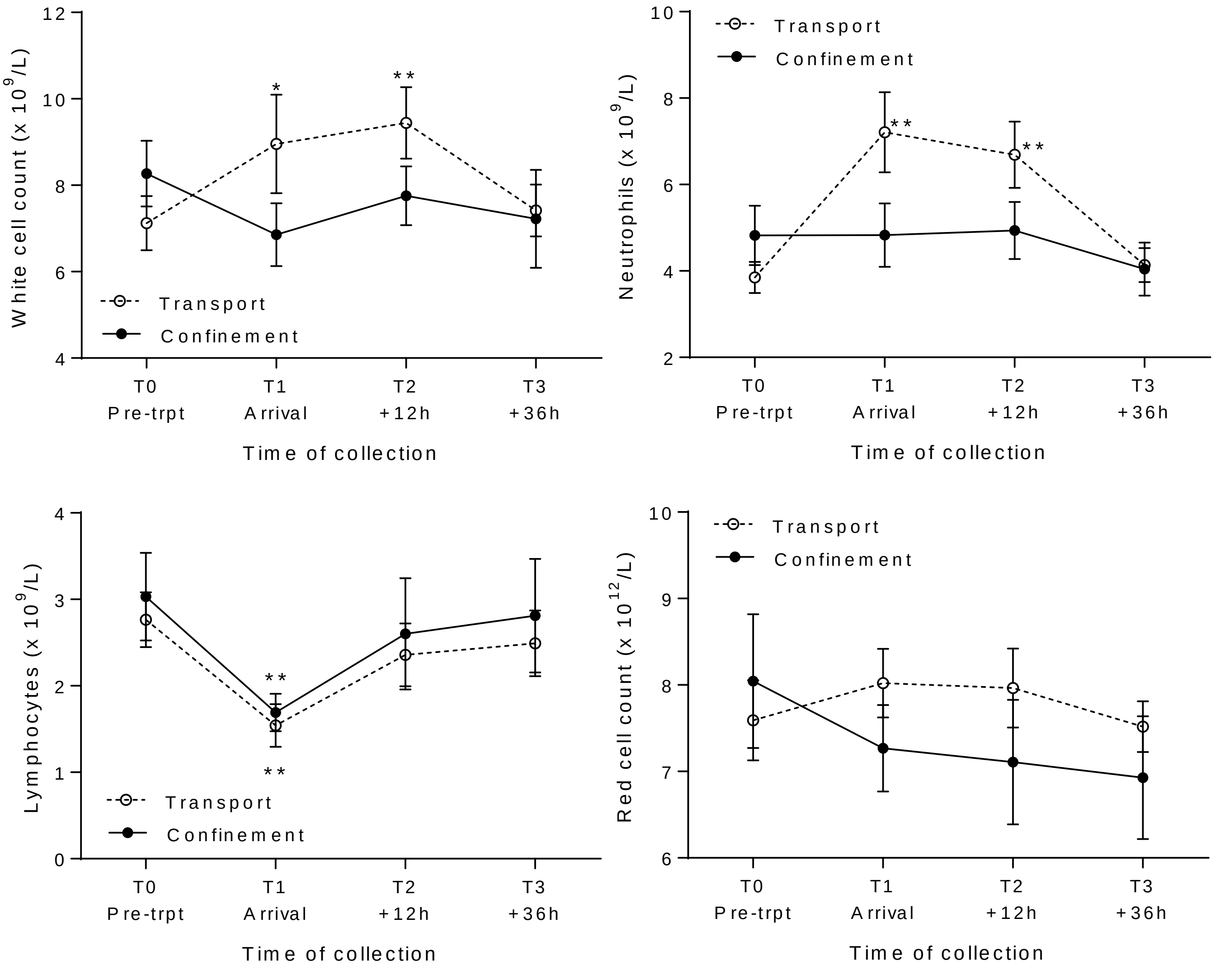

Figure S4: Effect confinement and transportation on plasma creatinine kinase, protein and albumin concentrations. Significant effects within each treatment are shown (P<0.05; **, P<0.005); P values refer to significant differences between transportation and confinement at specific time points. Results are shown as median and 95%CI for CK, and were log transformed for statistical analyses. Other results are presented as mean and 95% CI.

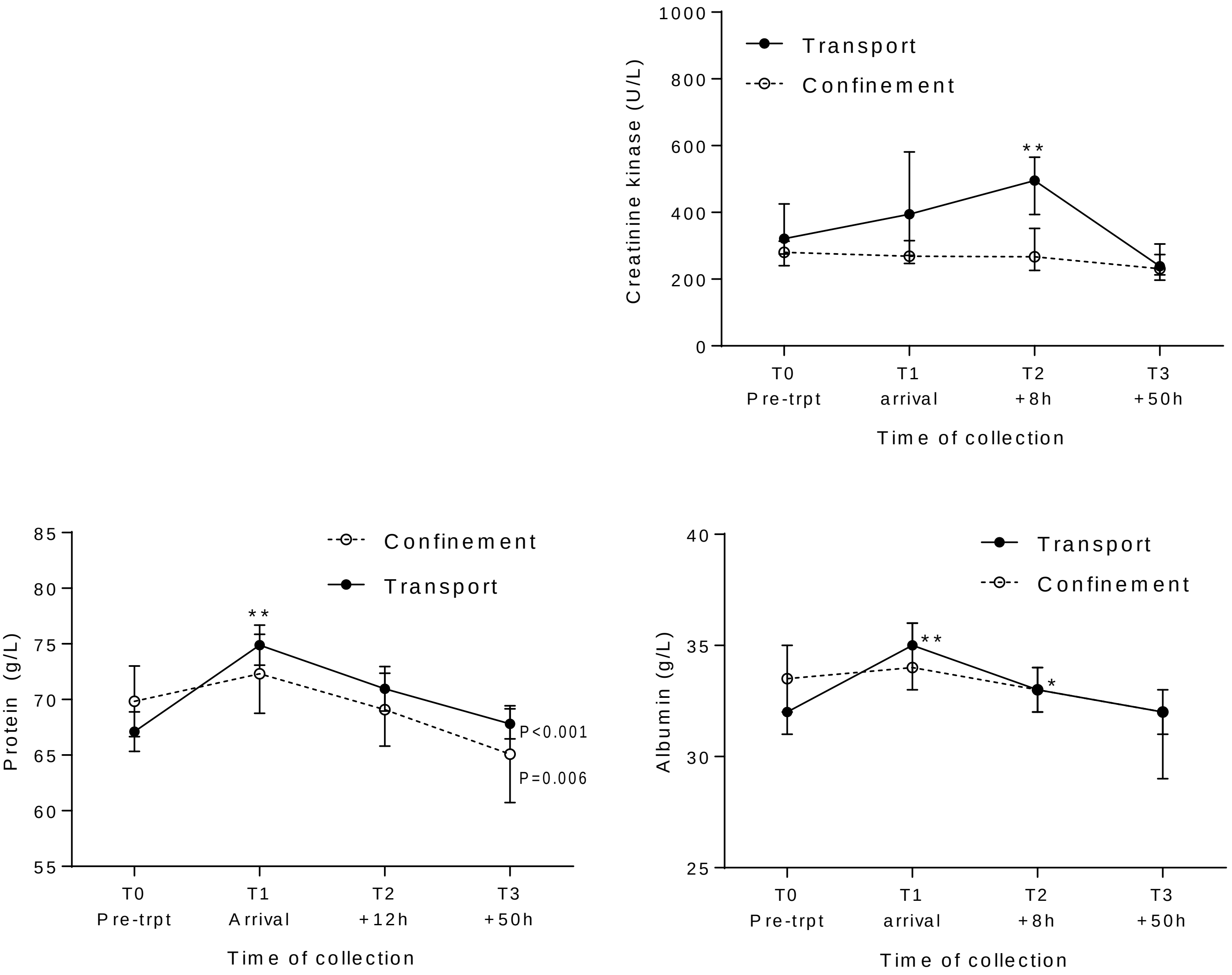

Figure S5: Sodium, potassium and calcium ion concentrations associated with transportation and confinement. Significantly differences in post-intervention results are shown (*, P<0.05; **, P<0.001). Sodium results are shown as median and 95% CI, and analyses were performed on log transformed data; other results are shown as mean and 95% CI. Plasma sodium increased slightly immediately after transportation and decreased subsequently such that differences observed at T2 and T3 were significantly less than observed as T1, as shown. Changes observed with confinement were not significant, and differences between confined and transported horses were not different at any time. Plasma potassium ion concentrations changed with time during both transportation and confinement, with significant differences between T1 and subsequent samples as shown. Differences in plasma potassium concentrations between transported and confined horses at T2 were significant (P=0.003). Plasma ionised calcium concentrations increased following transportation, but did not change associated with confinement. Differences between transported and confined horses were not significant at any time.

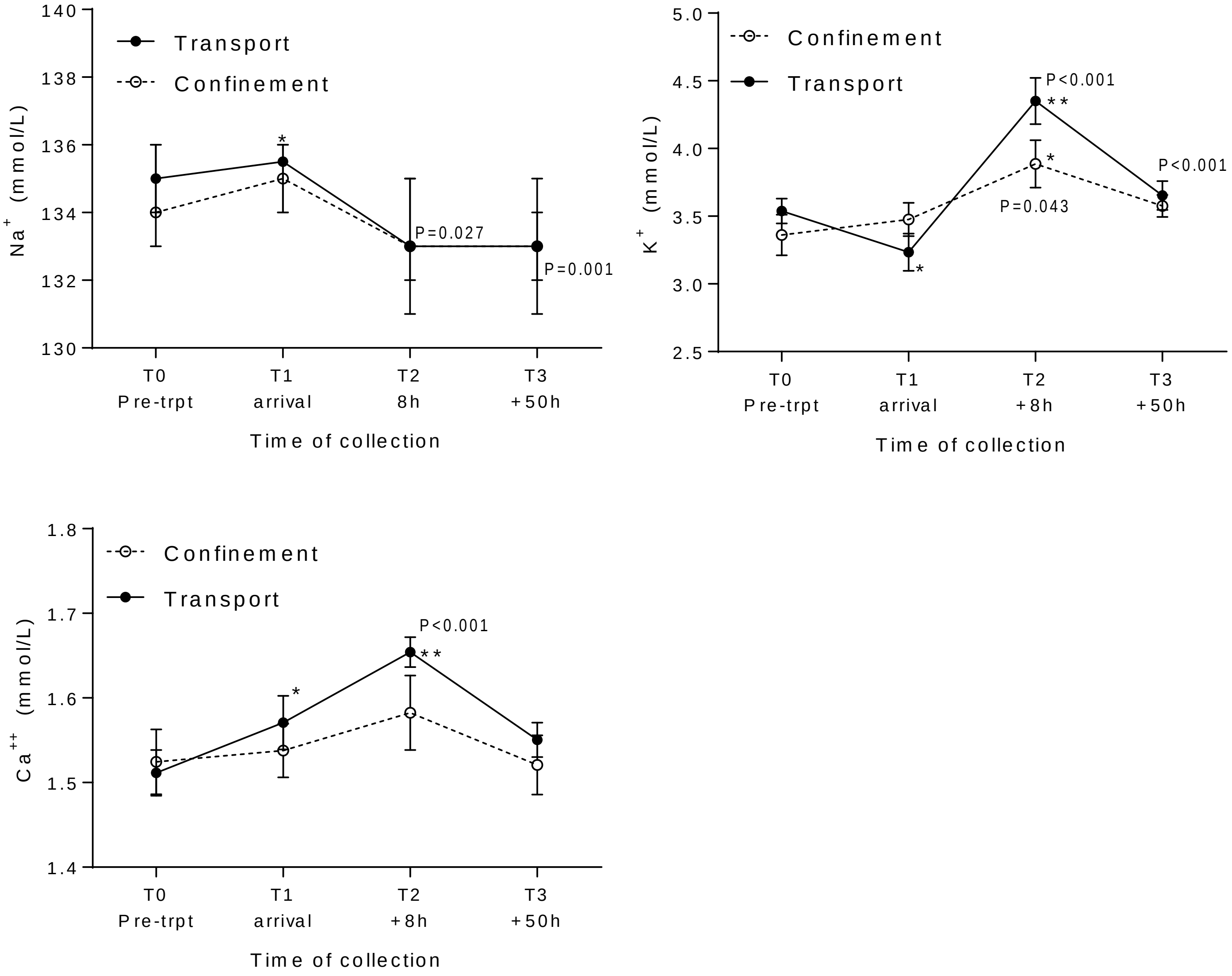

Figure S6: Plasma glucose and lactate concentrations associated with transportation and confinement. Significant differences between pre- and post-transport values are shown (**, P<0.001), and significant differences between T1 and T2, and between T1 and T3 are indicated. Differences in plasma lactate concentration in transported horses were different to those obtained associated with confinement at all time points (all P<0.001), and differences in plasma glucose concentration between transported and confined horses were significant at T1. Results are shown as median and 95% CI, and analyses were performed on log transformed data.

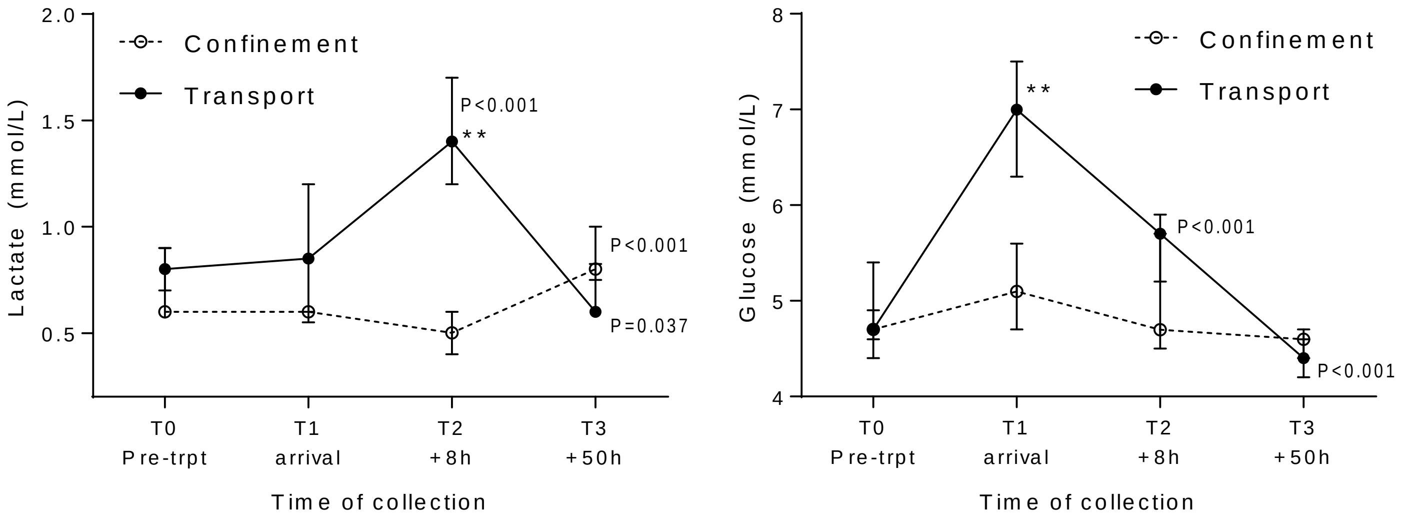

Figure S7: Grade 4 ulceration of the lesser curvature in two horses at T3, 60h following transportation.

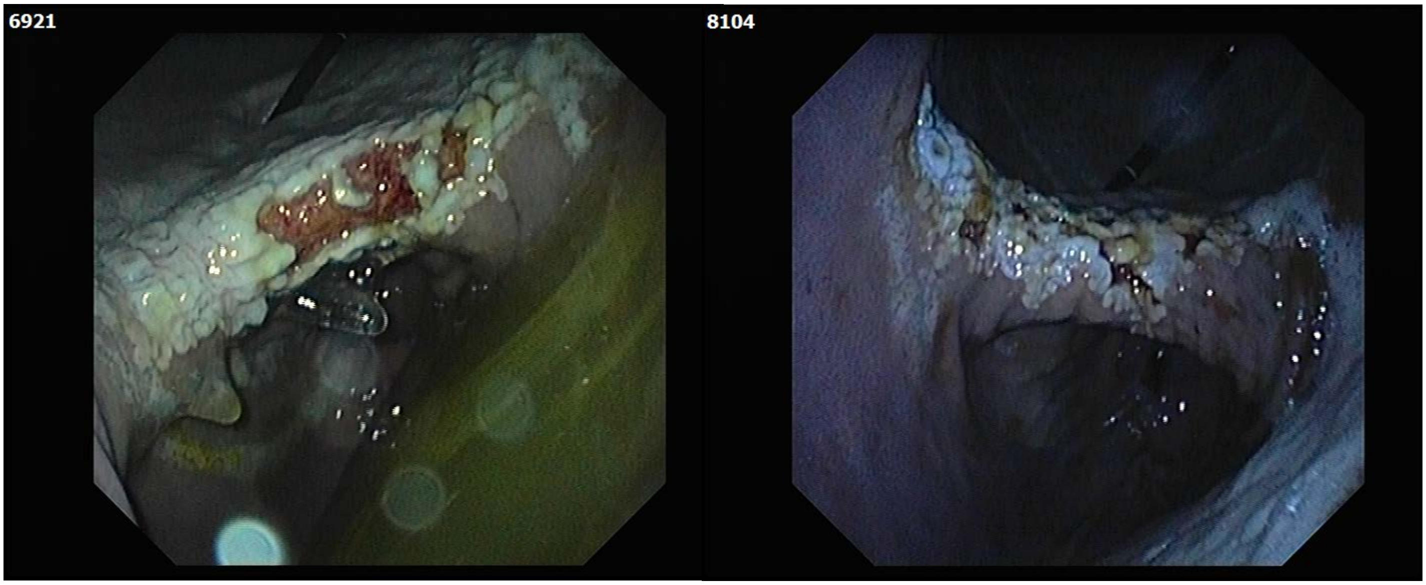

Supplement: Supplementary file 1 — Data S1: Details of study design Figure S1. Experimental design. Horses were recruited in 2 groups, each of 13 mares. From each group, a subset of 6 mares was randomly selected for a preliminary study to determine the effect of overnight (12 hours) confinement on gastric ulceration and pH of gastric fluid. Confinement occurred on 2 consecutive nights, with the 6 horses from each group confined separately according to available facilities. Prior to confinement, gastric squamous and mucosal ulceration was assessed by gastroscopy, and indwelling nasogastric tubes (NGT) were placed to allow sampling of fluid every 2 hours during the night. After a 14‐day break, the effects of transportation were assessed in 2 identical trips on consecutive nights. The 12 horses that underwent confinement were transported (6 horses in each consignment) with indwelling NGT in place. Sampling was identical for these 12 horses before confinement and transportation: all horses were fasted for 12 hours prior to gastroscopy and placement of NGT before confinement or transportation. In order to assess the effect of feeding prior to transportation, remaining horses in group 1 (n = 7, trip 1) were fed <60 minutes before transportation, and remaining group 2 horses (n = 7, trip 2) were fed 6 hours before departure. Of necessity (to allow feeding on the day of transportation), gastroscopy of these 14 horses was performed the day before departure (T‐1), and these horses did not travel with indwelling NGT. Pretransportation gastroscopy findings at T‐1 and T0 were combined for analysis. Clinical examination and venous blood collection were performed for all horses prior to confinement / transportation (T0), and repeated on completion of 12 hours (overnight) confinement / transportation at 0600h (T1). Gastroscopy was repeated for all horses on completion of confinement / transportation (T1), and took approximately 120 minutes to complete for all 13 horses following transportation. Gastric fluid was asp [file JVIM-34-922-s001.pdf]
